# Risk factors of esophageal fistula induced by re-radiotherapy for recurrent esophageal cancer with local primary site

**DOI:** 10.1186/s12885-022-09319-4

**Published:** 2022-02-24

**Authors:** Xinran Wang, Bing Hu, Jinhu Chen, Feihong Xie, Dan Han, Qian Zhao, Hongfu Sun, Chengrui Fu, Chengxin Liu, Zhongtang Wang, Haiqun Lin, Wei Huang

**Affiliations:** 1 Shandong Cancer Hospital and Institute, Shandong First Medical University and Shandong Academy of Medical Sciences, No.440, Jiyan road, Huaiyin distract, Jinan, 250117 Shandong Province China; 2 Department of Oncology, Jinxiang people’s hospital, Jinxiang, Shandong Province China

**Keywords:** Esophageal cancer, Esophageal fistula, Radiotherapy, Risk factor

## Abstract

**Purpose:**

The purpose of the present study was to investigate risk factors for esophageal fistula (EF) in patients with recurrent esophageal cancer receiving re-radiotherapy with or without chemotherapy.

**Methods:**

We reviewed retrospectively the clinical characters and dosimetric parameters of 96 patients with recurrent esophageal cancer treated with re-radiotherapy in Cancer Hospital Affiliated to Shandong First Medical University between August 2014 and January 2021.Univariate and multivariate logistic regression analyses were provided to determine the risk factors of EF induced by re-radiotherapy.

**Results:**

The median time interval between two radiotherapy was 23.35 months (range, 4.30 to 238.10 months). EF occurred in 19 patients (19.79%). In univariate analysis, age, T stage, the biologically equivalent dose in the re-radiotherapy, total biologically equivalent dose, hyperfractionated radiotherapy, ulcerative esophageal cancer, the length of tumor and the maximum thickness of tumor had a correlation with the prevalence of EF. In addition, age (HR = 0.170, 95%CI 0.030–0.951, *p* = 0.044), T stage (HR = 8.369, 95%CI 1.729–40.522, *p* = 0.008), ulcerative esophageal cancer (HR = 5.810, 95%CI 1.316–25.650, *p* = 0.020) and the maximum thickness of tumor (HR = 1.314, 95%CI 1.098–1.572, *p* = 0.003) were risk factors of EF in multivariate logistic regression analysis.

**Conclusions:**

The incidence of EF was significantly increased in patients with recurrent esophageal cancer who underwent re-radiotherapy. This study revealed that age, T stage, ulcerative esophageal cancer and the maximum thickness of the tumor were risk factors associated with EF. In clinical work, patients with risk factors for EF ought to be highly concerned and individualized treatment plans should be taken to reduce the occurrence of EF.

## Background

Loco-regional recurrence is the main type of failure in patients with esophageal cancer (EC) following chemoradiotherapy (CRT). Loco-regional recurrence is very common, occurring in approximately 40–60% of patients [1, 2]. Once recurrence occurs, most patients lost the chance of surgery [3, 4]. The prognosis of recurrent patients is poor and the mortality is high. Patients will die without treatment within 1 year [5]. The 5-year survival rate is only 0–11% [6, 7].

It is difficult to treat those patients with recurrent esophageal cancer (REC) after primary radiotherapy (RT). There are no general treatment guidelines for REC after primary RT. In patients with advanced REC, the effects of tumor recurrence are extremely distressing, and the main purpose of treatment is to relieve the patients’ dysphagia. Chemotherapy is a palliative treatment, which rarely achieves remission of the lesion. Re-radiotherapy (re-RT) appear to be an important treatment for local recurrence of EC after primary RT. The use of re-RT can significantly alleviate the symptoms of dysphagia, thereby improving the survival time and quality of life of patients [8].

The high incidence of complications of re-RT is a major problem especially esophageal fistula (EF), which is one of the serious complications. Anatomically, the esophagus is a muscular tube without serosa layer. Therefore, local extension of tumor to adjacent structure is common due to the lack of barrier to loco-regional spread such as the pericardium, trachea, mediastinum [9]. In addition, CRT can induce EF because of the imbalance between tumor shrinkage and normal tissue repair [10, 11]. EF can easily lead to serious infections, including pneumonia, lung abscess and sepsis. The mortality of patients with EF is high. Most patients with EF die within 3–4 months [12, 13]. Therefore, early prevention, early diagnosis and early treatment of EF are very important. The incidence of EF in EC patients receiving CRT has been reported to be 6–22% [14]. However, there are few reports on risk factors of EF caused by re-RT for REC patients. We conducted this study to answer this question.

## Materials and methods

### Patients’ selection

This study retrospectively analyzed 96 patients who were treated with re-RT in Cancer Hospital Affiliated to Shandong First Medical University between August 2014 and January 2021. The eligibility criteria were as follows: 1. All patients with pathologically confirmed REC with local primary site;2. Re-staged as II–IV based on the American Joint Committee on Cancer (7th edition);3. Karnofsky performance status (KPS) score ≥ 70;4. Treated by primary RT or re-RT with or without chemotherapy;5. The target area of primary RT and re-RT partially overlapped;6. Patients without any other serious medical illness except EC.7. No EF before re-RT. The exclusion criteria were as follows: 1. Patients underwent esophageal surgery previously; 2. Lost to follow-up. It should be noted that this study only included tumor recurrence in the primary tumor bed, with or without lymph nodes recurrence.

### Pretreatment evaluation

All patients underwent a physical examination, barium esophagography, fiber esophagoscopy, endoscopic ultrasonography, pathological and cytological examination, the cervical, chest and abdomen contrast-enhanced computed tomography (CT), magnetic resonance imaging (MRI) of the head. The diagnosis of recurrence after the primary RT for EC was based on pathological examination. The T stage was diagnosed by oncologists and radiologists based on findings of contrast-enhanced CT and endoscopic ultrasonography. The maximum thickness of the tumor was measured with MRI, CT or/and Positron Emission Tomography-Computer Tomography (PET-CT) by taking the maximum thickness of internal diameter and external diameter. The tumor length was determined by barium esophagography, esophagoscope, CT, MRI, or/and PET-CT. Esophageal stenosis is based on the patient’s clinical symptoms combined with the measurement results of barium esophagography or esophagoscopy. The time interval between two RTs was defined as from the end of primary RT to the beginning of re-RT.

### Treatment programs

All patients with REC included in the study were treated with concurrent CRT, sequential CRT or RT alone.

### Radiotherapy

All patients underwent re-RT. Each patient was placed in supine position with a body vacuum bag or head and neck thermoplastics technology, raising both arms and crossing elbows. The scanning range was from the ring membrane to 5 cm below the lower edge of the lungs, a slice thickness of 3.0 mm. The CT image was transmitted to the Varian planning system, radiologists and radiation oncologists collectively delineate the target area and the endangered organ. The gross tumor volume (GTV) included recurrent tumor lesions and metastatic lymph nodes that could be seen on CT/PET-CT/MRI. The clinical target volume (CTV) was subclinical lesions and high-risk lymphatic drainage areas [15]. The planning target volume (PTV) was defined as 0.5–0.8 cm beyond the CTV. Radiation was administered via a 6 MV X-ray, and 3 to 6 irradiation fields IMRT were used to pass the dose. The volume histogram was optimized, 95% isodose line covered the planned target area, 73 patients (76.04%) received conventional fractionated RT with the median dose of 50.4 Gy (16.0–61.2 Gy), 1.8–2.0 Gy / time, 5 times / week; 23 patients (23.96%) received hyperfractionated RT with the median dose of 50.4 Gy (31.2–60.0 Gy), 1.15–1.30 Gy / time, twice a day. Regarding the lungs, the V20 and mean dose were limited within 30% and 20 Gy respectively in the first treatment, after recurrence V20 was less than 25%. The highest dose of the spinal cord was < 25 Gy, and the mean dose of the heart was ≤30 Gy.

### Chemotherapy

Patients with REC generally chose the following two chemotherapy regimens: PF scheme include 5-fluorouracil (5-FU) 1000 mg/m^2^ on days 1–5 or S-1 60-80 mg/m^2^ on days 1–14 plus cisplatin (DDP) 25 mg/m^2^ on days 1–3. DP scheme include docetaxel (TXT) 75 mg/m^2^ or paclitaxel 135–150 mg on day one combined with DDP 25 mg/m^2^ on days 1–3. Both schemes were repeated every 21–28 days.

### Diagnostic criteria of EF

**Fig. 1:**
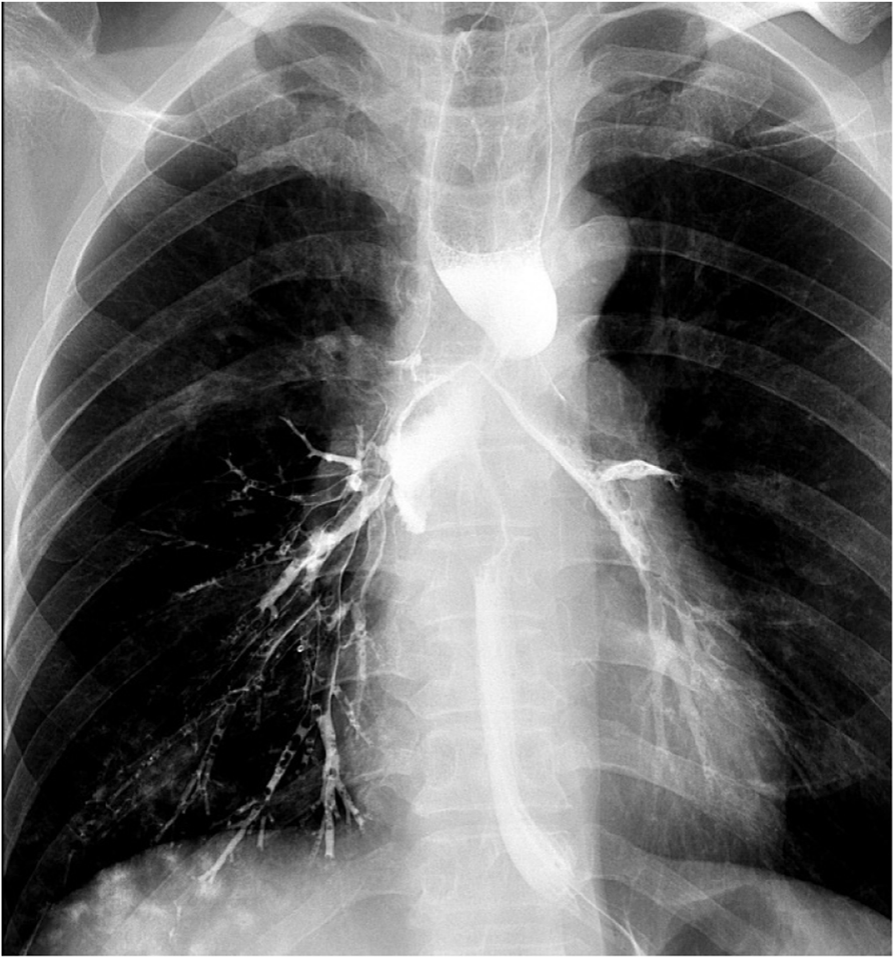
Esophagus barium meal examination shows esophago-respiratory fistula

**Fig. 2:**
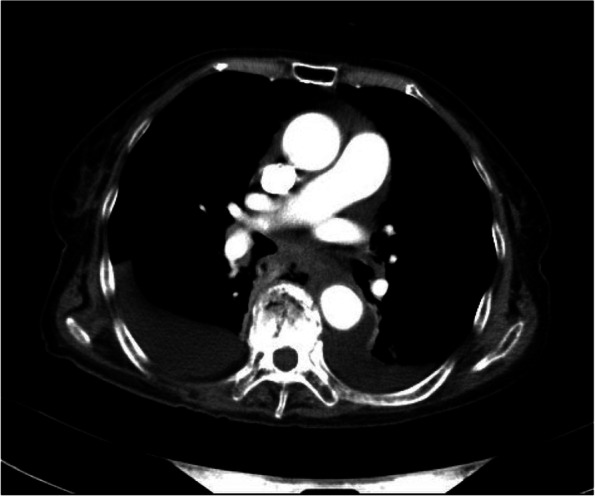
CT scan of the chest shows esophageal-mediastinum fistula

Common symptoms of EF include severe cough caused by consuming water or food, chest pain and fever. Discovery of fistulas by barium esophagography or/and esophageal endoscopy is the gold standard for the diagnosis of EF. Barium esophagography shows that the contrast medium entered the trachea, mediastinum or aorta through the fistula (see Fig. 1). CT is also an important method for the diagnosis of EF (see Fig. 2). Types of EF include esophageal-mediastinum fistula (EMF), esophago-respiratory fistula (ERF) and aorto-esophageal fistula (AEF). In this study, no patients developed AEF.

### Data collection

The following clinical characters and dosimetric parameters were collected and analyzed. Clinical characters include age, gender, location of the tumor and stage, the length of tumor, the maximum thickness of the tumor, esophageal stenosis, the time interval between two RTs, ulcerative EC, concurrent CRT in primary RT, concurrent CRT in re-RT. Dosimetric parameters include the biologically equivalent dose (BED) in re-RT, the total BED and hyperfractionated RT in re-RT.

### Statistical analysis

Retrospectively summarized and analyzed datum from all patients. The incidence of EF was calculated for all patients during or after RT. Univariate analysis was performed for 15 variables by logistic regression methods. Next, to select informative risk factors, the meaningful variables (*P*-value< 0.1) detected by univariate analysis were subjected to multivariate analysis. Univariate and multivariate analyses were carried out using logistic regression to estimate the odds ratio (OR) and 95% confidence intervals (CIs). *P*-value< 0.05 was considered statistically significant. All analyses were performed using IBM SPSS Statistics version 23.

### Follow-up

The last follow-up was in May 2021, and the median follow-up period was 14.80 months (range 0.33–90.83 months). The follow-up rate was 100% based on medical records, outpatient records, and telephone follow-up. Follow-up assessments were performed every 3 months in the first 2 year, followed every 6 months. At each follow-up visit, evaluation including physical examination, contrast-enhanced CT of the cervical region, chest, and abdomen and barium esophagography.

## Results

### Patient features

In this study, 96 patients were enrolled. EF was observed in 19 patients, and the incidence of EF was 19.79%. 3 patients developed EF during re-RT and 16 patients experienced EF after re-RT. The median time interval between the date of re-RT completion and EF diagnosis was 3.2 months (range, 0.6 to 9.3 months). The specific characteristics of patients were listed in Table 1.

**Table 1:** General clinical information of patients

Characteristics	Number of patients (*N* = 96)	Number of EF patients (*N* = 19)
Age (years)		
<70	59 (61.46%)	16 (84.21%)
≧70	37 (38.54%)	3 (15.79%)
Gender		
Female	23 (23.96%)	2 (10.53%)
Male	73 (76.04%)	17 (89.47%)
T stage		
Non-T4	65 (67.71%)	8 (42.11%)
T4	31 (32.29%)	11 (57.89%)
TNM clinical stage		
IIA-IIB	30 (31.25%)	5 (26.32%)
IIIA-IIIC	43 (44.79%)	6 (31.58%)
IV	23 (23.96%)	8 (42.1%)
Location of tumor		
Cervical section	10 (10.42%)	1 (5.26%)
Upper thoracic	38 (39.58%)	12 (63.16%)
Mid thoracic	32 (33.33%)	3 (15.79%)
Lower thoracic	16 (16.67%)	3 (15.79%)
Concurrent CRT in primary RT	30 (31.25%)	5 (26.32%)
Concurrent CRT in re-RT	26 (27.08%)	5 (26.32%)
Median BED in re-RT	59.47 (19.20–74.34)	54.00 (31.20–61.20)
Median total BED	131.47 (84.00–155.15)	135.72 (104.60–153.60)
Hyperfractionated RT in re-RT		
No	73 (76.04%)	18 (94.74%)
Yes	23 (23.96%)	1 (5.26%)
Ulcerative EC		
No	69 (71.88%)	10 (52.63%)
Yes	27 (28.12%)	9 (47.37%)
Esophageal stenosis (cm)		
<0.5	23 (23.96%)	3 (15.79%)
0.5–1	67 (69.79%)	15 (78.95%)
≥ 1	6 (6.25%)	1 (5.26%)
Median the length of tumor (cm)	4.35	5
The length of tumor (range) (cm)	(2–11)	(3–10)
Median the maximum thickness of tumor (mm)	14.685	17.24
The maximum thickness of tumor (range) (mm)	(7.17–29.61)	(12.75–29.61)
Median the time interval between two RTs (months)	23.35	24.4
The time interval between two RTs (range) (months)	(4.30–238.10)	(8.87–61.27)
Type of EF		
EMF		8 (42.11%)
ERF		11 (57.89%)
AEF		0

*EF* Esophageal fistula, *CRT* Chemoradiotherapy, *RT* Radiotherapy; *re-RT* re-radiotherapy, *BED* Biologically equivalent dose, *EC* Esophageal cancer, *EMF* Esophageal-mediastinum fistula, *ERF* Esophago-respiratory fistula, *AEF* Aorto-esophageal fistula

### Survival

**Fig. 3:**
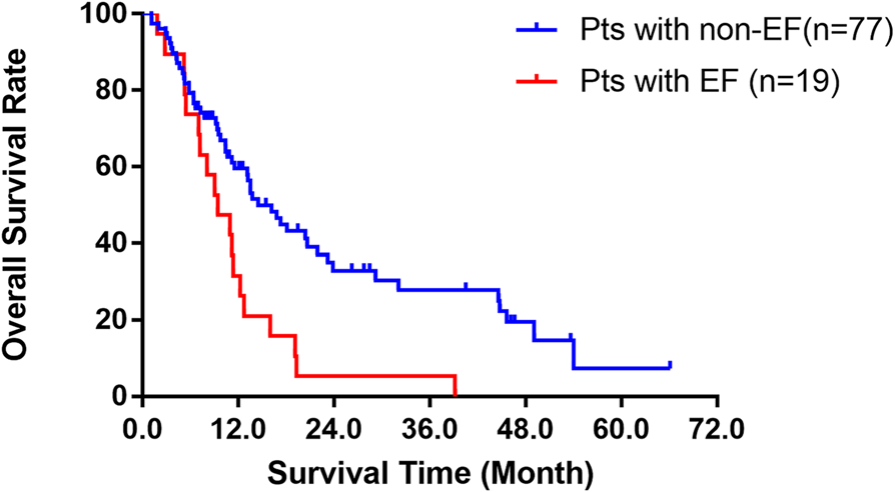
There was a significant difference between overall survival rates in patients with non-EF and in patients with EF (Kaplan-Meier method)

The Kaplan-Meier method was used to calculate the survival time from the first day of diagnosis of recurrence to the day of death, seen Fig. 3. Overall survival considered deaths from any cause. The median survival time (MST) of 77 patients with non-EF was 14.5 months (95% CI: 10.302–18.698), and the 6-month, 1-year and 2-year overall survival rates were 79.2,59.6 and 32.9%, respectively. The 6-month 1-year and 2-year overall survival rates in the 19 patients with EF were 73.7,31.6 and 5.3%, respectively, with an MST of 9.4 months (95% CI: 5.371–13.429). There was a significant difference between survival rates in the two groups (log-rank test, *p* = 0.0016). In the previous study of EC patients who underwent RT with or without chemotherapy [16], the MST of patients without EF and patients with EF were 36.8 vs 5.3 months, respectively. The prognosis of patients with EF was very poor, and all EF patients died during the follow-up period.

### Risk factors for EF

In the univariate analysis, age, T stage, the BED in re-RT, total BED, hyperfractionated RT in re-RT, ulcerative EC, the length of tumor and the maximum thickness of tumor were selected as meaningful factors for EF. The results of univariate analysis of risk factors for EF were shown in Table 2. The meaningful factors were included in multivariate analysis. Age, T stage, ulcerative EC and the maximum thickness of tumor had a significant correlation with the incidence of EF. The detailed information was shown in Table 3.

**Table 2:** Results of univariate analysis of risk factors for EF

Characteristics	EF-	EF+	OR	95%CI	*P*-value
Age (years)					
<70	43	16	1		
≧70	34	3	0.237	0.064–0.881	0.032
Gender					
Female	21	2	1		
Male	56	17	3.187	0.677–14.997	0.142
T stage					
Non-T4	57	8	1		
T4	20	11	3.919	1.380–11.126	0.010
TNM clinical stage					
IIA-IIB	25	5	1		
IIIA-IIIC	37	6	0.811	0.223–2.948	0.750
IV	15	8	2.667	0.736–9.665	0.135
Location of tumor					
Cervical section	9	1	1		
Upper thoracic	26	12	4.154	0.471–36.609	0.200
Mid thoracic	29	3	0.931	0.086–10.095	0.953
Lower thoracic	13	3	2.077	0.185–23.298	0.553
Concurrent CRT in primary RT	25	5	0.743	0.241–2.293	0.605
Concurrent CRT in re-RT	21	5	0.952	0.305–2.971	0.933
Median the BED in re-RT	59.47	54			
The BED in re-RT (range)	(19.20–72.0)	(31.20–61.20)	1.056	1.991–1.126	0.094
Median the total BED	131.47	135.72			
The total BED (range)	(84.0–155.15)	(104.60–153.60)	1.046	1.996–1.099	0.072
Hyperfractionated RT in re-RT					
No	55	18	1		
Yes	22	1	0.139	0.017–1.105	0.062
Ulcerative EC					
No	59	10	1		
Yes	18	9	2.95	1.039–8.378	0.042
Esophageal stenosis (cm)					
<0.5	20	3	1		
0.5–1	52	15	1.923	0.502–7.363	0.340
≥ 1	5	1	1.333	0.113–15.704	0.819
Median the length of tumor (cm)	4	5			
The length of tumor (range) (cm)	(2–11)	(3–10)	1.314	1.039–1.663	0.023
Median the maximum thickness of tumor (mm)	13.54	17.24			
The maximum thickness of tumor (range) (mm)	(7.17–24.87)	(12.75–29.61)	1.226	1.084–1.387	0.001
Median the time interval between two RTs (months)	23.33	24.4			
The time interval between two RTs (range) (months)	(4.30–238.10)	(8.87–61.27)	0.989	0.974–1.003	0.131

*EF* Esophageal fistula, *CRT* Chemoradiotherapy, *RT* Radiotherapy, *re-RT* re-radiotherapy, *BED* Biologically equivalent dose, *EC* Esophageal cancer, *CI* Confidence interval, *OR* Odds ratio

**Table 3:** Results of multivariate analysis of risk factors for EF

Characteristics	OR	95%CI	*P*-value
Age (years)			
<70	1		
≧70	0.170	0.030–0.951	0.044
T stage			
Non-T4	1		
T4	8.369	1.729–40.522	0.008
The BED in re-RT	1.063	0.918–1.231	0.416
The total BED	0.986	0.881–1.102	0.798
Hyperfractionated RT in re-RT			
No	1		
Yes	0.091	0.006–1.417	0.087
Ulcerative EC			
No	1		
Yes	5.810	1.316–25.650	0.020
The length of tumor (cm)	1.138	0.818–1.585	0.443
The maximum thickness of tumor (mm)	1.314	1.098–1.572	0.003

*RT* Radiotherapy, *re-RT* re-radiotherapy, *BED* Biologically equivalent dose, *EC* Esophageal cancer, *CI* Confidence interval, *OR* Odds ratio

## Discussion and conclusions

The local recurrence after primary RT in patients with EC is a tough challenge for clinical oncologists, it was as high as 66.5% after RT with or without chemotherapy in 2 years [17]. The vast majority of patients with REC have missed the opportunity for radical surgery, re-RT may be an effective modality [18]. The condition of some patients could be under long-term control, and the overall survival rate and survival rate after relapse could be improved. But EF is one of the serious complications, which is the main cause of treatment failure and death. The incidence for this event was reported to be 18–20% [3, 19]. In the same center, Xu et al. [20] reported that ECOG PS, BMI, T4, N2/3 and re-RT were independent factors for EF, then a nomogram was constructed and externally validated for the prediction of EF associated with RT. In our previous study [16], we also analyzed the risk factors associated with EF after RT for esophageal squamous cell carcinoma, it was found that T4 stage, N3 stage, re-RT, ulcerative EC, esophageal stricture and maximum tumor thickness were risk factors for EF. Among these factors re-RT was a strong risk factor for EF. Thus, we conducted this research to confirm the risk factors for EF in patients with REC receiving re-RT. In total, 15 clinical and dosimetric factors were included in the analysis. Age, T stage, ulcerative EC and the maximum thickness of tumor were revealed as risk factors for fistula formation.

Han et al. [21] reported that of 20 patients with EF, 14 of them were caused by RT. Esophageal perforation caused by RT is mainly due to the imbalance between the regression speed of tumor tissue and the repair speed of normal tissue. The rapid regression of tumor is related to the sensitivity of tumor for radiation, dose and speed of radiation. Kim et al. [3] reported that 17 patients with REC received re-RT, and 3 patients developed EF (17.65%). Zhou et al. [19] also reported on the efficacy and feasibility of salvage RT in patients with locally REC after radical CRT, this study showed that although re-RT could prolong the survival time of patients, the incidence of EF was as high as 20% (11/55). In our study, there were 19 patients with EF. The probability of EF in patients receiving re-RT was higher than that in patients receiving primary RT.

Esophagus tumor has a strong invasion to surrounding tissues and adjacent organs, which is related to the high incidence of EF [22]. Especially in T4 stage, the tissues and organs around the esophagus are more severely invaded. However, the esophagus surrounds the aorta, trachea, bronchus and mediastinum. The tumor can not only invade the esophageal wall, but invade the surrounding tissues and organs to form EF as well. The EF rates reported in T4 patients receiving CRT was in the range of 10–12% [14, 23]. In this study, the incidence of EF in T4 patients receiving re-RT was 57.89%, which greatly increased the risk of EF. Therefore, more attention should be paid to patients with T4 stage. Our results also found that the larger maximum thickness of the tumor was prone to EF. We analyzed that it might be related to the fact that the thickness of the tumor determined the irradiation area, which in turn affected the irradiation dose of important organs around the esophagus, leading to this serious complication. But the BED in re-RT and the total BED were not statistically significant in the occurrence of EF. For patients with REC, the suitable irradiation dose of remains uncertain, and further research is needed. We recommend that the total dose be as low as possible as higher dose was reported to increase the risk of perforation [24]. Our study revealed that the incidence of EF was relatively higher and statistically significant in patients with ulcerative EC than those with non-ulcerative EC. In the study of Tsushima et al. [25] 100% of patients with EF had ulcerative tumor. It was suggested that ulcerative EC was more prone to EF. Statistical analysis also showed that age<70 was a risk factor for EF. Compared with conventional RT, this study found that 23 patients underwent re-RT using hyperfractionated treatment modality, of which only 1 patient developed EF. However, whether hyperfractionation modality can reduce the incidence of EF needs to be verified in future randomized clinical surveys.

There were several limitations in this retrospective study including a smaller number of cases and the shorter follow-up period. Second, it was difficult to accurately distinguish between treatment-related EF and EF resulting from tumor progression, and finally, this was a study from a single center.

In conclusion, this study showed that age, T stage, ulcerative EC and the maximum thickness of the tumor were closely related to EF. Once EF occurs the prognosis is highly poor, no matter what kind of treatment strategy the effect is not good. Thus, the focus is on prevention. We should carefully formulate individualized treatment plans, highly select patients suitable for re-RT, strengthen adjuvant treatment, and minimize the risk of EF. In recent years, tumor immunotherapy has become a research hotspot of scholars at home and abroad. In the next study, we can explore whether immunotherapy combined with RT will increase the risk of EF.

## Data Availability

The datasets used and analyzed during the current study are available from the corresponding author on reasonable request.

## Abbreviations

EC: Esophageal cancer
CRT: Chemoradiotherapy
REC: Recurrent esophageal cancer
RT: Radiotherapy
re-RT: Re-radiotherapy
EF: Esophageal fistula
KPS: Karnofsky performance status
CT: Computed tomography
MRI: Magnetic resonance imaging
PET-CT: Positron Emission Tomography-Computer Tomography
GTV: Gross tumor volume
CTV: Clinical target volume
PTV: planning target volume
TXT: Docetaxel
DDP: Cisplatin
5-FU: 5-fluorouracil
EMF: Esophageal-mediastinum fistula
ERF: Esophago-respiratory fistula
AEF: Aorto-esophageal fistula
BED: Biologically equivalent dose
OR: Odds ratio
CIs: Confidence intervals
MST: Median survival time
